# Assessment of students’ perspectives about master of public health program in medical school of Shiraz University

**Published:** 2016-01

**Authors:** SAMAN FARAHANGIZ, ALIREZA SALEHI, RITA REZAEE, MOHAMMAD HADI IMANIEH

**Affiliations:** 1 MPH Department, Medical School, Shiraz University of Medical Sciences, Shiraz, Iran;; 2 Research Center for Traditional Medicine and History of Medicine, Medical School, Shiraz University of Medical Sciences, Shiraz, Iran;; 3 Quality Improvement in Clinical Education Research Center, Education Development Center, Shiraz University of Medical Sciences, Shiraz, Iran

**Keywords:** Students, Medical education, Public health, Satisfaction

## Abstract

**Introduction:**

Integration of public health and medical education has been thought to have an important role in medical students’ training. Shiraz University of Medical Sciences has developed an MD/MPH dual degree educational program for the talented volunteer students. The aim of this study was to assess the students’ viewpoints about various aspects of Shiraz MD/MPH program.

**Methods:**

This cross-sectional study was conducted on Shiraz undergraduate medical students, who were enrolled in MD/MPH program. A self-structured questionnaire in Persian consisting of 4 parts was used; it included demographic factors including 16 questions which evaluated the students’ perspective of the goals, content, skill development, applicability and meeting their expectations; 7 questions evaluating the self-reported increase of knowledge; and 3 multiple choice questions to assess the students’ motivations and opinions on the impact of the program on their future career. Descriptive statistics was used for data analysis.

**Results:**

All MD/MPH students (89) with a mean age of 21.4±1.34 participated in this study. Forty one of the students (46.1%) were male and 48 (53.9%) female. Overall, 86.1% of them had positive views about the goals of the program; also, 83.5%, 81.2% and 81.9% of them reported a positive viewpoint about the contents, the applicability and development of specific skills, and meeting their expectations, respectively. The students’ most frequent motivation was “learning how to research systematically” (73%). The majority of the students reported this program to be moderately to highly effective in increasing their knowledge in the provided courses.

**Conclusion:**

The students had a positive view about almost all of the aspects of the MD/MPH program; this may be indicative of the program being successful in delivering the goals, increasing the students’ knowledge and skills, and meeting their expectations to date. Students’ enthusiasm for the educational program may lead to their motivation for better learning and thus in the program’s success.

## Introduction

The substantial role of combining public health education in conventional medical education has obtained much attention (1, 2). Despite the effort to enter the public health requirements into the medical education curricula, still the social scope of health is not much focused in medical students’ training (3-5).

In this regard, Master of Public Health (MPH) program, which offers the essential competencies of public health education, was introduced in several universities in the world to be integrated with the medical degree (MD) training (6-12).

Since 2012, Shiraz University of Medical Sciences (SUMS) has developed an MD/MPH dual degree education program, in order to train the talented volunteer students the core implications of community-based health such as preventive medicine, advanced research methodology, communicable and non-communicable diseases epidemiology, health management, policy making and economics, and evidence-based health sciences (13).

We aimed to conduct this study to determine the students’ viewpoints about various aspects of MD/MPH program including the content, goal, application, and skill and knowledge development; we also attempted to use these views to improve the outcome and achievement of the objectives of the program. 

## Methods

This was a cross-sectional study conducted in February 2015 in MPH department of Shiraz medical school. There were three groups of medicine undergraduate students in MD/MPH educational course; based on their entrance years to the university, all of them entered the program in the late basic sciences period of medical education. Census method of sampling was used. Those who were a medical and MD/MPH student at the same time and had the consent to participate in the study were entered. 

A self-structured questionnaire in Persian was used for data collection; it consisted of 4 parts:

1) Demographic factors including age, gender, marital status and nativity.
2) 16 questions for assessment of students’ points of view about the MD/MPH program and the answers were in Likert scale with four parts including agree, partially agree, partially disagree and disagree and coded as 3 to 0, respectively. Questions 1-3 assessed the students’ perspective about the goals of the MD/MPH program, questions 4-8 were on the students’ view about the content provided by the MD/MPH course, questions 9-14 were about the students’ viewpoint about the applicability and development of specific skills, and questions 15-16 measured the students’ self-report on meeting their expectations.
3) Seven questions for evaluation of the students’ self-report about the increase in their knowledge in each course. The questions were in the format of “To what extent MD/ MPH courses has increased your knowledge in comparison with other students who did not enter the program”, and the answers were in 3 scales of low, moderate and high and coded as 0 to 2.
4) Three general multiple choice questions with the possibility for the participants to choose more than one answer measuring the most appropriate time, the students’ most important motivation for taking MPH course, and the possible impact of MPH course on their future career.

Validity of the questionnaire was confirmed by some experts in the field and reliability was assessed after a pilot on 20 students and Cronbach’s alpha of the part of the questionnaire for evaluating the students’ perspective was 0.88 and that for the section on evaluating their self-report of knowledge increase was 0.87.

The questionnaire was self-administered and anonymously completed. Informed consent of the participants was obtained before providing the questionnaires and after explaining the study’s objectives to them. 

SPSS version 14 was used for data analysis including descriptive statistics, which are expressed by mean±SD and frequencies shown by percentage. 

Ethics committee of Shiraz University of Medical Sciences approved this study. 

## Results

A total of 89 (all) MD/MPH dual degree program students were enrolled. All of the students (100%) responded to the questionnaires.  The number of first year MPH students was19 (21.3%), and 33 (37.1%) and 37 (41.6%) of the participants were second and third year students, respectively.  Mean age ± SD of the students was 21.4 ± 1.34, and 41 (46.1%) were male while 48 (53.9%) of the students were female. The majority of the participants (97.8%) were unmarried. Forty six (61.3%) students were native and were from Shiraz and 38.7% of them were from cities other than Shiraz or rural areas. Demographic characteristics of the participants are shown in Table 1.

**Table 1:** Demographic characteristics of the participants of Shiraz MD/MPH program evaluation study

**MPH group** **parameter**	**Gender N(%)**		**Nativity N(%)**		**Marital status N(%)**		**Age (Mean±SD)**
	**Male**	**Female**	**Native**	**Non-native**	**Single**	**Married**	
**MPH 1**	12(32.4%)	25(67.6%)	19(73.1%)	7(26.9%)	36(97.3%)	1(2.7%)	22.41±1.15
**MPH 2**	19(57.6%)	14(42.4%)	18(60%)	12(40%)	32(97%)	1(3%)	21.03±0.98
**MPH 3**	10(52.6%)	9(47.4%)	9(47.4%)	10(52.6%)	19(100%)	0(0.0%)	20.15±0.68
**Total**	41(46.1%)	48(53.9%)	46(61.3%)	29(38.7%)	87(97.8%)	2(2.2%)	21.4±1.34

The evaluation of the students’ views about the goals of the MD/MPH program shows that a mean of 86.1% of them had positive points of view as they chose “agree” or “partially agree” and an average of 13.9% had chosen “disagree” or “partially disagree”; their views were considered negative. The mean percentage of participants with positive views about the content of the MD/MPH courses was 83.5%. Respectively, 81.2% and 81.9% of the students were positive about the applicability of the program, development of specific skills, and the program meeting their expectations; and 91% of the participants recommended other medical students to attend MPH classes.

The self-reports about the effect of MD/MPH program on the students’ knowledge separately for each presented course are shown in Table 2.

**Table 2:** Self-report effect of MD/MPH courses on students’ knowledge

**Course name/ Impact magnitude**	**Low (NO. of students %)**	**Intermediate (NO. of students %)**	**High (NO. of students %)**	**Total (NO. of students %)**
**Research methodology**	6(6.7%)	51(57.3%)	32(36%)	89(100%)
**Basic statistics**	12(13.5%)	36(40.4%)	41(46.1%)	89(100%)
**Advanced statistics**	9(12.9%)	22(31.4%)	39(55.7%)	70(100%)
**Epidemiology of communicable diseases**	11(15.7%)	38(54.3%)	21(30%)	70(100%)
**Epidemiology of non- communicable diseases**	11(15.7%)	35(50%)	24(34.3%)	70(100%)
**Evidence-based health sciences**	7(10%)	31(44.3%)	32(45.7%)	70(100%)
**Scientific writing**	6(16.2%)	15(40.5%)	16(43.2%)	37(100%)

In our participants’ opinion, the most appropriate timing for taking MPH course was gradually during the years of medical school training, with 84 (94.4%) of them agreeing. Only 1 (1.1%) and 2 (2.2%) students thought that “residency”, and “after graduating from medical school and before residency” are the best time to attend this educational course, respectively.

The most frequent motivation for attending MD/MPH program was “learning how to research systematically” with 65 (73%) positive opinions and the second most frequent motivation was “learning new things and gaining knowledge in the field of non-clinical issues” (64%). See Table 3.

**Table 3:** Frequency of students’ motivation for entering the MD/MPH program

**NO.**	**What is your main motivation for entering into the MD / MPH? **	**Number of positive opinions (%)**
**1**	Learning how to research systematically	65(73%)
**2**	Learning new things and gain knowledge in the field of non-clinical issues	57(64%)
**3**	Having a strong academic resume	50(56.2%)
**4**	Contributing to development of health promotion programs in the community	47(52.8%)
**5**	Use this course to help to continue their studies abroad	29(32.6%)
**6**	Interest in the tasks of leadership responsibilities in health system	28(31.5%)
**7**	Competition with other students	12(13.5%)

MD/MPH program was thought to have a positive impact on the students’ future career as being a research physician (76.4%), being employed in research centers (36%), being in management responsibilities (24.7%) and positive effect on entering residency program (11.2%).

## Discussion

We evaluated the students’ views about various aspects of Shiraz MD/MPH program for the first time since the development of this program in SUMS. Generally, the majority of the students considered all the scopes of this program, including the goals, content, application, and skill development, as positive. We found that more than 90% of MD/MPH students are informed about the main goal and objectives of the program, while the majority of them believed that they were tailored to the community’s health demands, and met the students’ individual educational demands and their expectations, compared to other successful MPH programs in other universities, which also mostly met their students’ expectations (12).

Most of the participants believed that the content of the program has been successful in developing a deeper insight on the main competencies of MPH program so far, and also of the role and responsibilities of physicians in community health promotion. However, the real impact must be evaluated after the graduation of the students from MD/MPH program. This perceived role of MPH program in creating thoughts on community-based health was also expressed by the students of Tehran University of Medical Sciences’ MPH program (11). Also, Zwanikken et al. reported that the MPH graduates’ research was mostly on community health needs at their workplace after their graduation (14).

The high proportion of students with positive views about the applicability and skill development of the program can mean that they agreed that this program can be a good guide for them on becoming a competent professional person in their future jobs and can develop the ability to interact with various groups of members of society. Moreover, the majority of the students agreed that the program may have the potential to develop or improve skills such as critical thinking and articles reading, scientific writing and communication skills. Also, evaluation of the outcomes of MPH in a university in Canada showed that the majority of their students agreed with the improvement of their abilities across the core competencies (15).

Recommending MPH course to other medical students may show the success of the program in meeting the students’ expectations regarding their perception about the program goals based on the initial information given to them. This was also seen in other studies in different universities (11, 12).

We found that the vast majority of our students believed that all of the provided courses, specially research methodology, biostatistics, scientific writing and evidence-based health sciences have moderately to highly resulted in increasing their knowledge in these fields in comparison with other medical students, who have not attended the program; only a small percentage of them reported a low effect of the courses on their knowledge. Thus, this indicates that MD/MPH program may have been successful in both developing skills and increasing knowledge in these competencies although this should be evaluated by other measurement methods such as professors’ assessment in future studies. This impact of MPH program on increasing the knowledge and skills of students in public health related fields has been seen in the graduates’ future jobs in six countries by Zwanikken et al. (14).

Almost all of the students agreed that the most appropriate time for taking MD/MPH program is gradually during medical school training. This is in accordance with the time that we provide the program. This period of time during medical education for presenting MPH course was also agreed by Brown University MD/MPH students (12).

Learning how to research systematically, learning non-clinical issues in the field of medicine, and contribution to community health promotion were three most important motivations of our students for entering MD/MPH program. This indicates that students’ primary motivations and the courses offered in the program were somewhat parallel to each other that may have resulted in their positive views about the received training. But, interest in gaining leadership responsibilities in the future comprised less than one third of the students’ motivations, which may be due to lack of provision of health management and health policy making courses at the time of this study and being less familiar to these fields. Our students’ motivations were somehow similar to those of Tehran University program (11).

Most of the students believed that having an MPH degree may have positive impact on their future career as being a research physician and working in research centers and less thought that it would help them get a leadership responsibility. This was also in accordance with Tehran University students’ opinions (11). Also, Zwanikken et al. reported that MPH graduates had promoted their career to leadership positions and also doing research in community based health fields; this is similar to our students’ views (14).

The strength of our study is that we assessed Shiraz MD/MPH students’ points of view about all the aspects of the program and the results will help us improve this educational program and plan for the future to make the students more willing to achieve its main goals. There were also some limitations; we used a questionnaire with closed questions to assess the students’ perspective. Moreover, there are still no graduates in this program to evaluate the final impact. 

## Conclusion

Finally our students viewed almost all of the aspects of the MD/MPH program positively; this may be indicative of the program being successful in delivering the goals, increasing the students’ knowledge and skills, and meeting their expectations to date. On the other hand, students’ enthusiasm for the educational program and their satisfaction with it may lead to their motivation for better learning and thus in the program’s success. Future studies are recommended to be conducted to assess the students’ perspectives about recently presented training courses of the program over time. A cohort study for the comparison of the impact of MD/MPH on the students’ future careers may be done and compared with other medical students’ future jobs. Also a qualitative study can be conducted to better evaluate the students’ point of view. 

## Notes

**Financial Disclosure:** None declared.

## References

[1] Maeshiro R, Koo D, Keck CW (2011). Integration of public health into medical education: an introduction to the supplement. Am J Prev Med.

[2] Gillam S, Maudsley G (2010). Public health education for medical students: rising to the professional challenge. J Public Health (Oxf).

[3] Ruis AR, Golden RN (2008). The schism between medical and public health education: a historical perspective. Acad Med.

[4] Woodward A (1994). For Debate: Public health has no place in undergraduate medical education. J Public Health (Oxf).

[5] Horwitz RI A Proposal for Radical Reform of Medical Education [Internet].

[6] Harris R, Kinsinger LS, Tolleson-Rinehart S, Viera AJ, Dent G (2008). The MD-MPH program at the University of North Carolina at Chapel Hill. Acad Med.

[7] Cooper SP, McCormick JB, Chappell CL, Clare N, Vela L, Walker T (2010). Texas needs physicians trained in public health: a new 4-year integrated MD/MPH degree program. Tex Med.

[8] Imperato PJ, LaRosa JH, Schechter L (2005). The development of a Master of Public Health Program with an initial focus on urban and immigrant health at the State University of New York, Downstate Medical Center. J Community Health.

[9] (c2015). Stanford University [Internet].Stanford School of Medicine UC Berkeley Dual Degree MD-MPH Program.

[10] Sharma K, Zodpey S, Morgan A, Gaidhane A, Syed ZQ (2013). Designing the framework for competency-based master of public health programs in India. Public Health ManagPract.

[11] Manavi S, Nedjat S, Pasalar P, Majdzadeh R (2013). What Motivates Talented Medical Students to Study Simultaneously at Master of Public Health (MPH)?. Iran J Public Health.

[12] Smith SR (1996). Perceptions of the MD-MPH option at Brown. Acad Med.

[13] Salehi A, Hashemi N, Saber M, Imanieh MH (2015). Designing and conducting MD/MPH dual degree program in the Medical School of Shiraz University of Medical Sciences. J Adv Med Educ Prof.

[14] Zwanikken PA, Huong NT, Ying XH, Alexander L, Wadidi MS, Magaña-Valladares L (2014). Outcome and impact of Master of Public Health programs across six countries: education for change. Hum Resour Health.

[15] Britten N, Wallar LE, McEwen SA, Papadopoulos A (2014). Using core competencies to build an evaluative framework: outcome assessment of the University of Guelph Master of Public Health program. BMC Med Educ.

